# Nosocomial Bloodstream Infections in Brazilian Pediatric Patients: Microbiology, Epidemiology, and Clinical Features

**DOI:** 10.1371/journal.pone.0068144

**Published:** 2013-07-04

**Authors:** Carlos Alberto Pires Pereira, Alexandre R. Marra, Luis Fernando Aranha Camargo, Antônio Carlos Campos Pignatari, Teresa Sukiennik, Paulo Renato Petersen Behar, Eduardo Alexandrino Servolo Medeiros, Julival Ribeiro, Evelyne Girão, Luci Correa, Carla Guerra, Irna Carneiro, Carlos Brites, Marise Reis, Marta Antunes de Souza, Regina Tranchesi, Cristina U. Barata, Michael B. Edmond, Brazilian SCOPE Study Group

**Affiliations:** 1 Instituto de Oncologia Pediatrica – IOP/GRAAC, Sao Paulo, Brazil; 2 Hospital Israelita Albert Einstein, São Paulo, Brazil; 3 Universidade Federal de São Paulo (UNIFESP), São Paulo, Brazil; 4 Hospital 9 de Julho, São Paulo, Brazil; 5 Santa Casa de Porto Alegre, Porto Alegre, Brazil; 6 Hospital Conceição, Porto Alegre, Brazil; 7 Hospital de Base, Brasília, Brazil; 8 Hospital Walter Cantidio, Fortaleza, Brazil; 9 Hospital de Diadema, São Paulo, Brazil; 10 Santa Casa do Pará, Pará, Brazil; 11 Hospital Espanhol, Salvador, Brazil; 12 Hospital do Coração, Natal, Brazil; 13 Hospital da UNIMED, Natal, Brazil; 14 Hospital das Clinicas de Goiânia, Goiânia, Brazil; 15 Hospital do Rim e Hipertensão, São Paulo, Brazil; 16 Universidade Federal do Triangulo Mineiro, Uberaba, Minas Gerais, Brazil; 17 Virginia Commonwealth University, Richmond, Virginia, United States of America; UW Medical School, United States of America

## Abstract

**Background:**

Nosocomial bloodstream infections (nBSIs) are an important cause of morbidity and mortality and are the most frequent type of nosocomial infection in pediatric patients.

**Methods:**

We identified the predominant pathogens and antimicrobial susceptibilities of nosocomial bloodstream isolates in pediatric patients (≤16 years of age) in the Brazilian Prospective Surveillance for nBSIs at 16 hospitals from 12 June 2007 to 31 March 2010 (Br SCOPE project).

**Results:**

In our study a total of 2,563 cases of nBSI were reported by hospitals participating in the Br SCOPE project. Among these, 342 clinically significant episodes of BSI were identified in pediatric patients (≤16 years of age). Ninety-six percent of BSIs were monomicrobial. Gram-negative organisms caused 49.0% of these BSIs, Gram-positive organisms caused 42.6%, and fungi caused 8.4%. The most common pathogens were Coagulase-negative staphylococci (CoNS) (21.3%), *Klebsiella* spp. (15.7%), *Staphylococcus aureus* (10.6%), and *Acinetobacter* spp. (9.2%). The crude mortality was 21.6% (74 of 342). Forty-five percent of nBSIs occurred in a pediatric or neonatal intensive-care unit (ICU). The most frequent underlying conditions were malignancy, in 95 patients (27.8%). Among the potential factors predisposing patients to BSI, central venous catheters were the most frequent (66.4%). Methicillin resistance was detected in 37 *S. aureus* isolates (27.1%). Of the *Klebsiella* spp. isolates, 43.2% were resistant to ceftriaxone. Of the *Acinetobacter* spp. and *Pseudomonas aeruginosa* isolates, 42.9% and 21.4%, respectively, were resistant to imipenem.

**Conclusions:**

In our multicenter study, we found a high mortality and a large proportion of gram-negative bacilli with elevated levels of resistance in pediatric patients.

## Introduction

Nosocomial bloodstream infections (nBSIs) are an important cause of morbidity and mortality and are the most frequent type of nosocomial infection in pediatric patients [1]–[4]. The crude mortality is high, particularly for critically ill patients [2], [3], [4]. In the ICU setting children with BSI had crude mortality rates of 52% in an Israeli hospital [2] and 14% in a multicenter study of US hospitals [1].

In addition to an increase in incidence of nBSI, the proportion caused by multidrug resistant pathogens is also rising [5]. Despite advances in antimicrobial treatment, nBSI prolongs hospital stay, increases direct patient care costs and directly causes mortality [6], [7].

The rates of antimicrobial resistance among pathogens causing healthcare associated infection are increasing, mainly among gram-negative organisms (*Pseudomonas aeruginosa*, *Acinetobacter baumannii* and *Klebsiella pneumoniae*) [8]. Indeed this increase in antimicrobial resistance demonstrates a need for surveillance programs to define the species distribution and the resistance patterns of pathogens that can cause a nBSI in order to help physicians choose the most appropriate antimicrobial treatment for hospitalized patients [9].

There are differences in surveillance programs in different parts of the world with regards to methodologies and populations studied (e.g., ICU, neutropenic, or haemodialysis patients) [9], [10], [11]. There are only a few studies focusing on nBSI in pediatric patients [1], [2]. The purpose of this study was to evaluate the epidemiological features of nBSI, the species distribution and the antimicrobial susceptibility of the pathogens in pediatric patients, using the same methodology of prior studies (US and Brazilian SCOPE Projects) [9], [12].

## Methods

The Brazilian Surveillance and Control of Pathogens of Epidemiologic Importance (Br SCOPE), based at Universidade Federal de Sao Paulo, Sao Paulo, Brazil, was established to identify the predominant pathogens and their antimicrobial susceptibilities of nosocomial bloodstream isolates [12]. The 16 participating hospitals throughout the five different regions in Brazil (North, Northeast, Middle-East, Southeast, and South), represent medical institutions of public and private hospitals. The study was approved by the Institutional Review Board (IRB) at each participating site and it was submitted to the final approval of the IRB from Universidade Federal de Sao Paulo. The requirements for informed consent was waived by IRB from Universidade Federal de Sao Paulo in accordance of the Code of Federal Regulations and of the Privacy Rule.

### Study Design

Clinical data and bloodstream isolates were prospectively collected by local infection-control practitioners using a standardized case-report form and forwarded to the coordinating center along with each microbiological isolate. We did not perform any training or data validation as the local infection control practitioners were experienced in performing surveillance. Patients admitted between June 12, 2007 and March 31, 2010 to any of the 16 hospitals participating in the Br SOPE Project were eligible if they were ≤16 years of age or treated on a pediatric service and met the criteria for a nosocomial bloodstream infection (BSI). For some descriptive analyses patients < = 1 year old and >5 years old were compared.

A nosocomial BSI was determined to have occurred if ≥1 culture of a blood sample obtained ≥48 hours after admission to the hospital yielded a pathogenic organism. If the bloodstream isolate was a potential skin contaminant (e.g., diphtheroids, *Propionibacterium* species, *Bacillus* species, coagulase-negative staphylococci, or micrococci), all of the following criteria were required for the diagnosis: the presence of an intravascular catheter, the initiation of targeted antimicrobial therapy, and at least one clinical finding (temperature >38.0°C or <36°C, chills, or systolic blood pressure of <90 mmHg). BSI episodes that represented relapses were excluded. We analyzed the data from all eligible episodes of BSI.

The data that were routinely collected included the patient’s age, gender, predisposing clinical conditions, species distribution and antimicrobial susceptibility of causative pathogens and outcome. Predisposing clinical conditions that were routinely recorded included neutropenia (defined as an absolute neutrophil count of <1000/µL), peritoneal dialysis or hemodialysis, and presence of intravascular catheters (i.e., central lines, arterial catheters, or peripheral intravenous catheters). Secondary BSIs were regarded as those with a clear source of bacteremia other than a central line. Sources of secondary BSI were identified by cultures of samples (urine, tracheal secretions, intra-abdominal samples, etc.) obtained form distant sites that yielded the same pathogen with an identical resistance pattern. Distant sites were sites where an infection was diagnosed other than a central line (pneumonia, urinary tract infection, abdominal infection, etc). Crude mortality was defined as in-hospital mortality. We also compared pediatric patients hospitalized at private vs. public facilities.

### Microbiological Methods

Blood cultures were processed at the participating hospitals. The identification of blood isolates and susceptibility testing were done with the routine methods in use at the affiliated laboratories. All affiliated laboratories were Brazilian Society of Clinical Pathologists-certified, and all microbiological methods used were consistent with current CLSI recommendations [13]. Data from all hospitals were used for analysis, and denominators for individual antimicrobial agents may vary because not all hospitals test and report all drugs.

At the reference laboratory, Special Microbiology Laboratory from Universidade Federal de São Paulo, samples identified by manual methods were submitted to re-identification and antimicrobial susceptibility test by the Phoenix BD automated system. MIC determination by E-TEST for oxacillin, and carbapenems and agar dilution for vancomycin were done to confirm resistant phenotypes.

Molecular tests were applied to resistant strains as follow:

*For Staphylococcus aureus*: *mec*A detection, SCC*mec* characterization [14], [15] and molecular typing by pulsed field electrophoresis (PFGE);
*Enterococcus* spp: v*an*A and *van* B detection
*Klebisiella pneumoniae*: ESBL detection (*bla*_TEM_, *bla*_CTX_, *bla*_SHV_) and carbapenemase detection (*bla*_KPC_)

### Statistical Analysis

The results were expressed as the mean± SD or as a proportion of the total number of patients or isolates. For continuous variables, mean values were compared using two sample t tests for independent samples. Differences in proportions were compared using a chi-square test or Fisher’s exact test, as appropriate. All tests of significance are 2-tailed; α was set at 0.05. All statistical analyses were done using SPSS software (SPSS).

## Results

### Study Population and Patient Characteristics

During the study period a total of 2,563 cases of BSI were reported by hospitals participating in the Br SCOPE project. Among these, 342 clinically significant episodes of BSI (13.3%) were identified in pediatric patients (≤16 years of age). Patients had a mean age of 4.7±5.1 (range, 0 to 16 years), but one-half of the patients were < = 1 year old (172/342, 50.2%).

At the time of diagnosis of BSI, nearly half of patients were hospitalized in a pediatric or neonatal intensive care unit (155; 45.3%). Others were in a general pediatric unit (71; 21.0%) or a pediatric hematology-oncology unit (60; 17.5%). Underlying conditions were classified as malignancy in 95 patients (27.8%), followed by respiratory in 54 patients (15.8%), and gastroenterology in 36 patients (10.5%). However, one-fifth of the patients (n = 65; 19.0%) were hospitalized secondary to conditions classified as “other”. These could not be analyzed further because of the lack of a classification of conditions seen in pediatric patients only. Thirty-eight patients (11.1%) were neutropenic at the time of BSI diagnosis.

Among the potential factors predisposing to BSI, intravascular devices were the most frequent. Central venous catheters were in place in 227 patients (66.4%), followed by peripheral iv catheters in 79 patients (23.1%) and arterial catheters in 9 patients (2.6%). A total of 61 patients were receiving parenteral nutrition (17.8%), and in 20 (5.8%) dialysis was needed at the onset of BSI. Ventilator support was necessary in 130 patients (38.0%). Overall 74 patients died during hospitalization, accounting for a crude mortality of 21.6%.

### Microbiologic Features

During the study period 357 isolates were recovered from 342 episodes of BSI. Of these, a total of 175 (49.0%) were caused by gram-negative organisms, 152 (42.6%) by gram-positive organisms; and 30 (8.4%) fungi, of which 27 (90.0%) were *Candida* species (table 1).

**Table 1:** Epidemiologic and demographic data of pediatric patients with nBSI.

Patient demographics	N (%)
**Total pediatric patients**	342
**Mean Age (±SD), in years**	4.7 (±5.1)
**Gender**	
Male	176 (51.5%)
Female	166 (48.5%)
**ICU setting**	172 (50.4%)
**Monomicrobial infection**	328 (95.9%)
**Organisms (N = 357)**	
Gram-negative	175 (49.0%)
Gram-positive	152 (42.6%)
Fungi	30 (8.4%)
**Underlying conditions**	
Malignancy	95 (27.8%)
Respiratory	54 (15.8%)
Gastrointestinal	36 (10.5%)
Other	65 (19.0%)
**Potential risk factors**	
Central venous catheter	227 (66.4%)
Ventilator	130 (38.0%)
Urinary catheter	76 (22.2%)
**Crude mortality**	74 (21.6%)

The rank order of the major pathogens (Table 2) shows that coagulase-negative staphylococci (CoNS), accounted for almost one-quarter of all nosocomial BSI (21.3%), followed by *Klebsiella* species (15.7%), *Staphylococcus aureus* (10.7%), *Acinetobacter* species (9.3%) and *Candida* species (7.6%). Four percent of all episodes of BSI (n = 13) were polymicrobial. In these, the most frequently isolated pathogens (n = 15) were *Klebsiella* species (33.3%), *Candida* species (20.0%), *Enterococcus* species (13.3%) and *Enterobacter* species (13.3%).

**Table 2:** Rank order of nosocomial bloodstream pathogens in pediatric patients among 16 hospitals throughout Brazil.

	Total		% of Isolates			Crude Mortality*
Organisms	No of isolates	% of isolates	Age <1 yr	Age 1–5 yr	Age>5 yr	(%)
Coagulase-negative staphylococci	76	21.3	50.0	21.1	19.0	26.3
*Klebsiella* species	56	15.7	–	19.6	13.8	17.6
*Staphylococcus aureus*	38	10.6	18.2	11.3	9.5	21.1
*Acinetobacter* species	33	9.2	–	8.3	13.8	25.0
*Candida* species	27	7.6	13.7	6.7	8.6	58.3
*Enterobacter* species	25	7.0	–	10.3	2.6	21.7
*Enterococcus* species	21	5.9	13.7	3.4	9.5	30.8
*Pseudomonas aeruginosa*	14	3.9	–	5.4	2.6	14.3
*Escherichia coli*	14	3.9	–	4.4	4.3	21.4
*Streptococcus* species	12	3.4	–	2.9	5.2	16.7
*Serratia* species	6	1.7	–	2.0	1.7	50.0

* Crude mortality of patients with monomicrobial BSI.

When different age groups were compared, the proportion of CoNS decreased from 50% in patients <1 year to 19% in patients >5 years old. The same pattern was seen with *S. aureus, Candida* species and *Enterococcus* species in the same patient populations decreased from 18%, 13% and 13%, respectively, to 9% for each. The proportion of *Acinetobacter* species was higher in patients >5 years old.

In patients with monomicrobial BSI (n = 328), the crude mortality (Table 2) ranged from 14% (for *Pseudomonas aeruginosa*) to 58% (for *Candida* species). Mortality in patients with polymicrobial BSI was 38.5%. Considering facility type, 90.9% (298/328) had nBSI at a public hospital and 9.1% (30/328) had nBSI at a private hospital. There was no statistically significant difference with regard to gender (p = 0.44), age (p = 0.68) and potential risk factors, such as the central venous catheter (p = 0.62), ventilator (p = 0.16), or urinary catheter (p = 0.40) between pediatric patients at a private or a public facility. There was also no statistically significant difference in crude mortality in pediatric patients at private (13.8%, 4/29) vs. public facilities (23.5%, 65/277; p = 0.24).

More pediatric patients acquired nBSI in ICUs in private facilities (70%, 21/30) than public (48.3%, 144/298), p = 0.024. Considering the organisms, in private facilities there were 66.7% (20/30) gram positive nBSIs, 30.0% (9/30) gram negative nBSIs, and only 3.3% (one case) of fungal nBSI, while in public facilities there were 49.0% (146/298) gram negative nBSIs, 42.6% gram positive nBSIs and 8.4% (25/298) fungal nBSI, p = 0.039.

Of the 27 *Candida* isolates causing nosocomial BSI, *Candida albicans* was responsible for 37% of cases of *Candida* BSI, followed in rank order by *Candida tropicalis* (26%) and *Candida parapsilosis* (22%). *Candida glabrata* accounted for only 4% of cases of *Candida* BSI.

### Antimicrobial Susceptibility

Methicilllin resistance was detected in 37 *S. aureus* isolates (27%) and in 76 CoNS isolates (92%). *S. aureus* strains with reduced susceptibility to vancomycin were not detected. Reduced susceptibility to teicoplanin was reported in 1 CoNS isolate (3%) (table 3).

**Table 3:** Rates of antimicrobial resistance among gram-positive organisms most frequently isolated from children with nosocomial bloodstream infection.

	*Staphylococcus aureus*		CoNS		*Enterococcus* spp	
Antimicrobial drug	No. of isolates	% resistant	No. of isolates	% resistant	No. of isolates	% resistant
Ampicillin	ND	–	ND	–	19	36.8
Methicillin	37	27.1	76	92.1	ND	–
Cefazolin	25	16.0	28	92.9	ND	–
Vancomycin	37	0	76	0	19	21.0
Teicoplanin	7	0	34	2.9	7	0
Linezolid	26	0	70	0	16	0
Ciprofloxacin	24	16.7	41	56.1	5	0
Clindamycin	35	22.9	72	73.6	ND	–
Gentamicin	36	19.4	73	60.3	17	11.8

CoNS = Coagulase-negative staphylococci; ND = Not done.

Thirty-five CoNS were characterized at species level: *Staphylococcus epidermidis* (62.8%), followed by *Staphylococcus hominis* and *Staphylococcus warneri* (11.4%), *Staphylococcus haemolyticus* (8.5%) and *Staphylococcus capitis* (5.7%). The susceptibility profile revealed high resistance rate to oxacilin (80%).

Twenty isolates of *Staphylococcus aureus* were studied at the reference laboratory and 5 of them were resistant to oxacilin. Molecular analysis was performed showing 4 *mec*A positive isolates. The characterization of SCC*mec* revealed 2 SCC*mec* type III isolates and one SCC*mec* type IVa.

For enterococci, vancomycin resistance was found in 19 isolates (21.0%), 80% of these were *E. faecium* isolates (N = 5) and none were *E. faecalis* strains (N = 8). Eleven *Enterococcus* spp were studied at the reference laboratory, 6 *E. faecalis* and 5 *E. faecium*. In only 3 *E. faecium* strains was the vancomycin MIC>256 µg/mL and the *van*A gene was detected in all 3.

Antimicrobial resistance levels for the most common gram-negative organisms causing nosocomial BSI are shown in table 4. Relatively high proportions of *Klebsiella* spp displayed resistance to ampicillin-sulbactam, piperacillin-tazobactam, ceftazidime, and cefepime (60.7%, 40.5%, 38.3%, and 37.7%, respectively). Resistance to imipenem and meropenem was seen in 2.0% and 1.9% of the isolates.

**Table 4:** Rates of antimicrobial resistance among gram-negative organisms most frequently isolated from children with nosocomial bloodstream infection.

	*Klebsiella pneumoniae*			*Acinetobacter baumannii*			*Enterobacter species*			*Pseudomonas aeruginosa*		
Antimicrobial drug	No. of isolates	% resistant	No. of isolates		% resistant	No. of isolates		% resistant	No. of isolates		% resistant	
Amp-Sul	28	60.7	24		33.3	ND		–	ND		–	
Pip-Tazo	37	40.5	18		55.6	22		36.4	12		25.0	
Cefazolin	ND	–	ND		–	ND		–	ND		–	
Ceftriaxone	44	43.2	ND		–	ND		–	ND		–	
Ceftazidime	47	38.3	27		55.6	24		29.2	13		38.5	
Cefepime	53	37.7	28		50.0	26		7.7	12		25.0	
Imipenem	51	2.0	28		42.9	26		0	14		21.4	
Meropenem	52	1.9	29		37.9	24		0	13		23.1	
Ciprofloxacin	52	15.4	29		37.9	25		0	14		21.4	
Gentamicin	47	32.7	27		25.9	25		8.0	12		25.0	

ND = Not done; Amp-Sul = ampicillin-sulbactam; Pip-Tazo = Piperacillin-tazobactam.

Eleven out of 39 *Klebsiella pneumoniae* isolates were phenotypically characterized as ESBL with the presence of genes *bla*_TEM_, *bla*_CTX_, *bla*_SHV_ in 3 (28.3%), 7 (63.6%) and 5 (45.4%), respectively. One isolate, resistant to imipenem and meropenem (MIC = 8 µg/mL) was modified Hodge test negative. The molecular analysis by polymerase chain reaction did not reveal the presence of the *bla*_KPC_ gene.

For *Acinetobacter* spp, cephalosporins, fluoroquinolones, and carbapenems were not active against almost 40% of the isolates tested. For *Enterobacter* species, nearly 40% were resistant to piperacillin-tazobactam. All *Enterobacter* species were susceptible to fluoroquinolones and carbapenems. Of the *P. aeruginosa* isolates, almost one-quarter were resistant to cephalosporins, aminoglycosides, fluoroquinolones, piperacillin-tazobactam and carbapenems.

## Discussion

National surveillance studies focusing on nBSI are important tools that can detect specific issues related to antimicrobial resistance [9], [10], [11], [12]. In addition, BSI surveillance studies are preferable because rigid and standardized clinical diagnostic criteria make data more reliable and realistic, avoiding the problem of confounding colonizing agents not directly related to clinical disease [9], [12]. BSIs are also the most serious and potentially life-threatening infectious diseases in the pediatric population and in the majority of cases antimicrobial therapy must be initiated empirically [1], [2], [3]. For these reasons accuracy in predicting the pathogens and the resistance profile is crucial for successful therapy.

Brazil is a country with more than 150 million inhabitants and a total surface area larger than 8,500,000 Km^2^. In addition to its large geographic size, the country is heterogeneous in sociodemographic indicators, with wealthier regions concentrated in the southern part of the country. Although the majority of the population depends on the public healthcare system, an increasing fraction is being managed by private facilities. Heterogeneity is also reflected in healthcare practices at the level of nosocomial infection management. As a result, different patterns of antimicrobial resistance and antimicrobial use may emerge within the country [12]. Also, due to its intrinsic characteristics, etiologic agents and their respective antimicrobial susceptibilities may differ from those of other countries [9], [10].

In this study, we analyzed 342 nBSI episodes in pediatric patients that were prospectively collected at 16 Brazilian medical centers between 2007 to 2010. This is the largest study done in the pediatric population in this country and it is important because epidemiological and resistance data of the adult population are frequently extrapolated to children. The knowledge of Brazilian data is of paramount importance for determining regional and national prevention and treatment strategies for nosocomial infections. Different from other previous nationwide network surveillance studies, the Br SCOPE Study [12], similar to the US SCOPE Study [9], is representative of the whole country, obtaining data from its 5 different geographical regions. Also, standardized criteria for BSI were applied, conferring reliability for comparison with other studies.

The crude mortality was 21% in this pediatric cohort, half of that observed in the adult population evaluated by the Br SCOPE [12], but higher than that reported by the US SCOPE Project (14%) [9]. However, US SCOPE (9) analyzed more ICU patients and fewer oncologic patients. We suspect that the higher mortality rate observed in our study was due to higher resistance levels in the gram negative bacilli. There was no difference in mortality rates between pediatric patients with nBSI in public or private facilities.

When we analyzed by pathogen, higher mortality was observed in *Candida* species (58.3%). This has been reported in other studies and may be due to the severity of the illness and the greater time to positive blood cultures, which accounts for a delay in the start of therapy. Another important finding of this study is the high mortality rate observed in patients with polymicrobial bacteremia (38%), possibly related to the difficulties in therapeutic management.

Like other studies of nBSI and/or healthcare related infections in Latin America [10], [12], we observed a higher prevalence of gram-negative bacilli over gram-positive cocci (49% *vs* 42.6%, respectively), whereas the US SCOPE found that only 24% of infections were due to gram-negative bacilli [9]. Of note, in the adult Br SCOPE population, the proportion of infections due to gram- negative bacilli was even higher (58.5%) [12]. This high prevalence of gram-negative bacilli in Brazil has a practical importance, especially when dealing with empiric treatment of suspected BSI infection since our data suggest that an antimicrobial agent directed against gram-negative pathogens is mandatory. We observed a higher proportion of gram negative nBSI in public hospitals (49%) than in private hospitals (30%) in our pediatric population.

It must be emphasized that in our study, in children younger than one year old, half of the nBSI were caused by coagulase negative staphylococci (CoNS) with an associated elevated crude mortality (26.3%), unlike that observed in other studies [16], [17] and this fact may be related to the high severity of infections in this age group. In the US SCOPE [9] it also was observed that many infections were caused by these agents in children less than one year old, but with only 10% mortality, which is what is expected for these agents and has been reported by other authors [16], [17].

High levels of resistance were observed in *Klebsiella pneumoniae:* 40% to cephalosporins (3^rd^ and 4^th^ generations) and piperacillin/tazobactan, when compared to data from US SCOPE [9], where only 10% were resistant to ceftazidime and 30% to piperacillin. However, a high level of susceptibility to carbapenems was observed in both studies. Although high, the resistance profile to cephalosporins was smaller than that observed in the Br SCOPE [12]. In this study, no carbapenamase-producing organisms were found and the majority of ESBLs were due to gen *bla*_CTX_.

Regarding *Pseudomonas aeruginosa*, US SCOPE [9] found a low level of resistance (<10%) to the majority of antibiotic agents. In our study, the resistance was greater than 20% for all antibiotics.

The worst resistance was observed with *Acinetobacter baumanni*: more than 55% were resistant to piperacillin/tazobactan and cephalosporins (3^rd^ and 4^th^ generations), 40% were resistant to carbapenems and ciprofloxacin. However, a greater level of resistance was observed in the adult Br SCOPE population (approximately 60% to carbapenems and 70% to cephalosporins and ciprofloxacin) [12]. These data confirm the high and worrisome level of resistance among gram-negative bacilli in Brazil, and the high prevalence of these agents may have a serious impact on antibiotic management, creating a vicious cycle of heavy use and resistance development.

Antibiotic resistance among the gram-negative bacteria is worrisome, especially regarding *P. aeruginosa* and *Acinetobacter baumanii*. Rates as high as 40% resistance to carbapenems for *Acinetobacter* and 23% for *P. aeruginosa* are among the highest in the world’s literature in the pediatric population and have several implications for clinical practice [15], [18]. Since resistance to carbapenems usually is associated with multi-drug resistance [19], very few options remain viable. In some instances, polymyxins remain the last alternative [20] and these drugs already widely used in Brazil [21] could be considered in empiric therapy for the subgroup of patients prone to develop BSI due to these agents.

Fungal nBSI has become more frequent. We found a prevalence of 8.4%, a slightly lower than that found in US SCOPE (9.3%) [9] and higher than that observed in adult Br SCOPE (6.1%) [12]. Possibly, it is due to a greater concentration of oncologic patients. Moreover, 11% of these patients were neutropenic at the time of BSI diagnosis.

In this study, species of *Candida* represented 7.6% of nBSI, the majority caused by non-albicans species (63%), mainly *C. tropicalis* and *C. parapsilosis*, similar to data from adult patients [12].

In conclusion, our study describes the pattern of nBSI in Brazilian children. We found a higher mortality in comparison to data from the US [9] and a larger proportion of gram-negative bacilli with elevated levels of resistance. This finding may aid Brazilian physicians in the empirical treatment of BSI in pediatric patients.
